# Benefits of the Enhanced Recovery After Surgery (ERAS) Pathway With Quadratus Lumborum Blocks for Minimally Invasive Gynecologic Surgery Patients: A Retrospective Cohort Study

**DOI:** 10.7759/cureus.49183

**Published:** 2023-11-21

**Authors:** Paul S Lee, Laurie L Brunette, Intira Sriprasert, Mohamed Eloustaz, Rasika Deshpande, Crystal Adams, Laila Muderspach, Lynda Roman, Shane Dickerson, Michael P Kim

**Affiliations:** 1 Anesthesiology, University of Southern California, Los Angeles, USA; 2 Obstetrics and Gynecology, University of Southern California, Los Angeles, USA; 3 Obstetrics and Gynecology, Los Angeles County Medical Center and University of Southern California Medical Center, Los Angeles, USA

**Keywords:** peri-operative analgesia, regional anesthesiology, chronic and acute pain management, enhanced recovery pathways (eras), oncological gynecology

## Abstract

Study objective: This study aimed to determine the effect of the implementation of the Enhanced Recovery After Surgery (ERAS) protocol among patients receiving minimally invasive gynecologic surgery.

Design and setting: This retrospective cohort study was performed in a tertiary care hospital.

Patients: A total of 328 females who underwent minimally invasive gynecologic surgeries requiring at least one overnight stay at Keck Hospital of University of Southern California (USC), California, USA, from 2016 to 2020 were included in this study.

Interventions: The institutional ERAS protocol was implemented in late 2018. A total of 186 patients from 2016 to 2018 prior to the implementation were compared to 142 patients from 2018 to 2020 after the implementation. Intraoperatively, the ERAS group received a multimodal analgesic regimen (including bilateral quadratus lumborum (QL) blocks) and postoperative care geared toward a satisfactory, safe, and expeditious discharge.

Measurements and main results: The two groups were similar in demographics, except for the shorter surgical time noted in the ERAS group. The median opioid use was significantly less among the ERAS patients compared with the non-ERAS patients on postoperative day 1 (7.5 vs. 14.3 mg; p<0.001) and throughout the hospital stay (17.4 vs. 36.2 mg; p<0.001). The ERAS group also had a shorter median hospital length of stay compared to the non-ERAS group (p<0.01). Among patients with a malignant diagnosis, patients in the ERAS group had significantly less postoperative day 1 and total opioid use and a shorter hospital stay (p<0.01). Within the ERAS group, 20% of the patients did not end up receiving a QL block. Opioid use and length of stay were similar between patients who did and did not receive the QL block.

Conclusions: The ERAS pathway was associated with a reduction in opioid use postoperatively and a shorter length of hospital stay after minimally invasive gynecologic surgery. There was a more significant decrease in opioid use and hospital length of stay for patients with malignant diagnoses compared to patients with benign diagnoses. Further research can be done to fully delineate the effect of QL blocks in ERAS protocols.

## Introduction

Over the past two decades, there has been an increased focus on improving the perioperative experience for surgical patients. One such vehicle, Enhanced Recovery After Surgery (ERAS), seeks to decrease both opioid intake and hospital length of stay. This pathway was initially developed for colorectal surgeries [1] and has since been implemented for a wide array of surgical subspecialties. ERAS has since become well established in the field of gynecologic surgery [2-7].

ERAS is a compilation of evidence-based, best-practice guidelines applied across the perioperative period to promote recovery and manage the metabolic stress response to surgery. Key preoperative ERAS components include addressing topics, such as limiting the time spent fasting, carbohydrate loading, and pain control. Intraoperatively, emphasis is placed on utilizing multimodal analgesia while taking measures to avoid postoperative oversedation. Postoperatively, priorities are multimodal pain control, early mobilization, and enteral feeding. The overall goal ultimately is a quick and safe discharge from the hospital.

Regional anesthesia has played a large role in minimizing opioid usage during the perioperative period for patients. The quadratus lumborum (QL) block targets the thoracolumbar nerves in the thoracic paravertebral space, providing both somatic and visceral analgesia to the abdominal wall and peritoneum [8,9]. Studies have also compared QL blocks against transversus abdominis plane (TAP) blocks for minimally invasive gynecologic surgeries and Cesarean deliveries [10-14] , demonstrating superior analgesia with QL compared to TAP blocks.

We hypothesized that an institution-wide implementation of an ERAS pathway with QL blocks would lead to a shorter length of hospital stay and decreased use of narcotic medications.

## Materials and methods

Study design

This was a retrospective cohort study of female patients in Keck Hospital of USC (Los Angeles, California), a tertiary-referral academic center undergoing laparoscopic or robotic gynecologic surgeries requiring at least one overnight inpatient stay. We reviewed institutional gynecologic surgery databases and included all patients who met these criteria from March 2016 to December 2020. Patients who underwent a small Pfannenstiel incision to remove large specimens intact at the end of the minimally invasive surgery were included. Patients whose surgeries were fully converted to laparotomy or those with chronic opioid use were excluded from the study. All patients were operated on by one of nine surgeons throughout the course of the study, including gynecologic surgeons, gynecologic oncologists, and urogynecologists. The study protocol was approved by the Institutional Review Board (IRB) of the University of Southern California (dated May 11, 2019), and the requirement for written consent has been waived.

There were no prior existing standardized perioperative practices for gynecological surgeries at our institution. An ERAS protocol was developed by a multidisciplinary perioperative team consisting of gynecologic surgeons, anesthesiologists, and nurses and was implemented for all patients undergoing laparoscopic or robotic gynecologic surgery starting in November 2018. The historical controls (non-ERAS group) included all sequential patients at our institution undergoing minimally invasive gynecologic surgery with at least one overnight hospital stay from March 2016 to October 2018. The ERAS group included all sequential patients undergoing minimally invasive gynecologic surgery with at least one overnight hospital stay from November 2018 to December 2020 that were automatically enrolled in the ERAS pathway at the time of scheduling their surgery. The use of narcotic medications was reported as the sum of opioid use on postoperative day 0, postoperative day 1, and the total use until discharge, and all narcotic medication doses were converted to morphine milligram equivalents (MMEs). The length of hospital stay was the time of arrival to the preoperative area - about two hours prior to surgery - until discharge (days). Our primary outcome was the total MME, while our secondary outcomes were MME on postoperative day 0 and 1 along with the length of hospital stay.

ERAS protocol intervention

Before implementing these interventions, the clinic, operating room, inpatient unit, and intensive care unit staff were educated on the benefits of an ERAS protocol and the specifics of the new interventions. Plain-language summary packets were created and distributed to the patients. Patients were given ERAS education at multiple points of contact, including in the surgery clinic, pre-anesthesia clinic, on the postoperative ward, at the time of discharge, and during follow-up phone calls to answer questions and reinforce teaching. Educational sessions for our staff were repeated on a quarterly basis, and open feedback forums took place to address questions and concerns.

Patients in the ERAS group were administered a preoperative analgesic regimen prior to surgery, which consisted of acetaminophen 1 g, gabapentin 300 mg, and celecoxib 200 mg (unless the patient had a history of chronic kidney disease or peptic ulcer disease) in the preoperative holding area immediately prior to surgery. Preoperative carbohydrate loading consisted of two bottles of Ensure® Pre-Surgery Clear Carbohydrate Drink (Abbott, USA) (50 g carbohydrates per bottle) that was given to patients at cost as a part of a pre-anesthesia clinic visit. Patients were advised to drink these two hours prior to arrival to the hospital, approximately four hours prior to anesthesia start time.

The induction and maintenance of anesthesia was per the anesthesia attending physician of the case following the intraoperative ERAS protocol (Appendix 1). Of note, several antiemetics (oral aprepitant 40 mg, scopolamine patch 1 mg, dexamethasone 10 mg, ondansetron 4 mg, and propofol infusion) were administered prior to and after induction to help mitigate the risk of postoperative nausea and vomiting. After induction of general anesthesia and prior to surgical incision, a QL block was performed by or under the supervision of an anesthesiologist familiarized with the ERAS protocol. While in the supine position, two blanket pads approximately 6 inches in diameter were placed under the patient’s hip and thoracic cage to scan the posterolateral aspect of the patient. A low-frequency curved array ultrasound transducer was placed in the transverse axis between the patient's iliac crest and costal margin with the depth set to 9 cm. The QL muscle was identified immediately deep to the transversus abdominis aponeurosis and attached to the L4 lumbar transverse process. After sterilization with chlorhexidine, a 20-gauge 100 mm echogenic needle was inserted in an anterior-to-posterior direction. The QL nerve block was performed by injecting local anesthetic between the QL and psoas muscles. The injectate was 20 mL 0.25% bupivacaine followed by 10 mL 1.33% liposomal bupivacaine on each side, administered in 5 mL aliquots with negative aspiration for blood and air between each aliquot. No further local anesthesia was injected at the incision sites if a patient received the QL block.

Postoperatively, the patients were ordered for a clear liquid diet immediately upon arrival to post-anesthesia care unit (PACU). Once this was tolerated for one meal, they were advanced to an "ERAS diet" that is a modified regular diet aimed at decreasing gas production by eliminating milk, raw fruits and vegetables, and high-fat foods. The urinary catheter was removed at six hours postoperatively, and early ambulation was encouraged. Postoperative analgesia included scheduled oral acetaminophen 1000 mg every eight hours and either intravenous ketorolac 15 mg every eight hours or oral ibuprofen 600 mg every eight hours around the clock. Oral tramadol 50 mg every six hours was given as needed for breakthrough pain. Intravenous morphine was given only if the patient was not tolerating oral or if oral pain medications were ineffective. Patients were discharged with acetaminophen 1000 mg and ibuprofen 600 mg every six to eight hours for three to five days and tramadol 50 mg every six hours as needed. Additional narcotics could be prescribed at the time of discharge on an individual patient basis if felt necessary by the surgeon, but these instances were rare (Appendix 2).

Sample size calculation and statistical analysis 

Demographic and clinical characteristics were summarized with descriptive statistics. Patient age, body mass index (BMI), American Society of Anesthesiologists (ASA) class, comorbidities, diagnosis of malignancy, surgical approach, type of surgery, and surgical time were compared between the ERAS and non-ERAS groups. Categorical variables were reported as frequency (percent) and compared between the ERAS and non-ERAS groups with a chi-squared test, while continuous variables were reported as mean (standard deviation (SD)) and compared with a t-test.

The Wilcoxon rank-sum test was used to compare median opioid use (postoperative day 0, postoperative day 1, and total opioid use) and length of hospital stay (days). The analysis was stratified by diagnosis (benign vs. malignant) and surgical approach (laparoscopic vs. robotic surgery). Among patients with the ERAS protocol, the opioid use and length of stay were further compared between patients with and without QL blocks.

Generalized linear models evaluated the association between ERAS status with log-transformed opioid use and length of stay adjusting for age and surgical time. Type of diagnosis and type of surgery were tested for the potential modifying effect on the association between ERAS status with log-transformed opioid use and length of hospital stay. Data from the medical chart were extracted and recorded in a Research Electronic Data Capture (REDCap), a Health Insurance Portability and Accountability Act of 1996 (HIPAA) compliant, secure, web-based database application for research study. All analyses were conducted in Stata Statistical Software release 16 (StataCorp., 2019, College Station, TX: StataCorp LLC). P-value less than 0.05 is considered as statistical significant. This manuscript adheres to the applicable Strengthening the Reporting of Observational Studies in Epidemiology (STROBE) guidelines.

## Results

**Figure 1:**
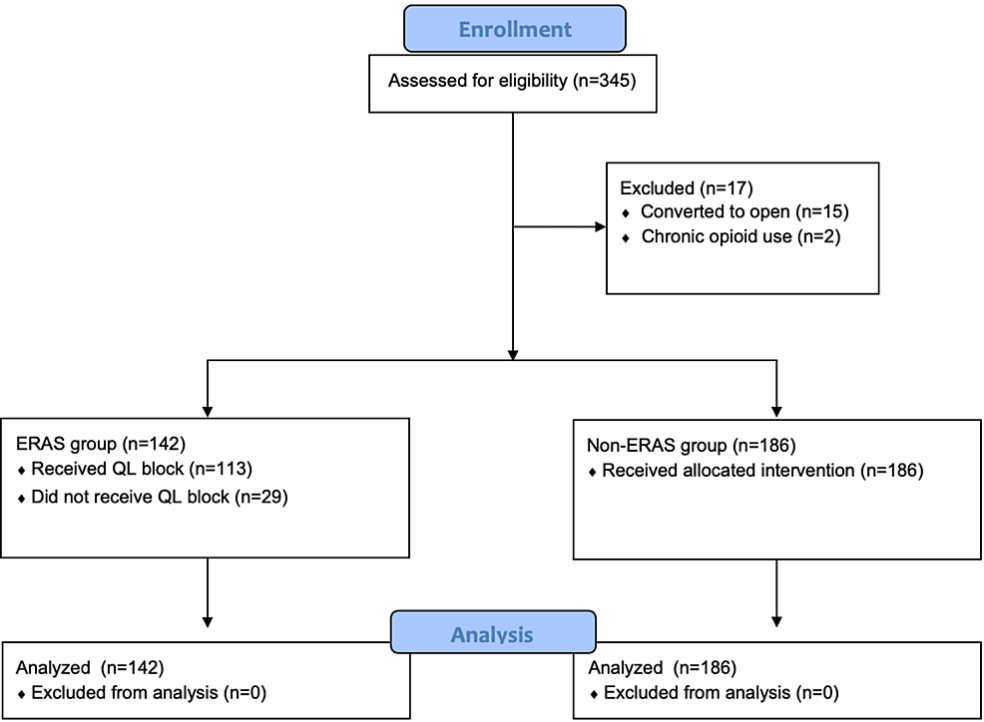
CONSORT flow diagram ERAS: Enhanced Recovery After Surgery, QL: quadratus lumborum

A total of 345 patients were evaluated for inclusion in this study, but 15 were excluded because their minimally invasive surgery was converted to laparotomy, and two were excluded for chronic opioid use (diagnosed fibromyalgia and myofascial pain syndrome). A total of 328 patients were included in the analysis; 142 patients were in the ERAS group and 186 patients were in the non-ERAS group (Figure 1). 

Demographics

Overall, the mean age (SD) of the participants was 51 (0.8) years and mean BMI was 28.5 (0.4) kg/m^2^. The demographics of the study population, including age, BMI, ASA class, comorbidities, diagnosis, surgical approach, surgeon specialty, and type of surgery, were similar between patients within the ERAS and non-ERAS groups. Most patients (73.5%) were diagnosed with benign pathology, and more than half of the patients (59.4%) underwent robotic surgery. The surgical time was significantly shorter among patients with ERAS compared to those not receiving the ERAS protocol: 235.5 (8.1) vs. 263.3 (7.3) minutes; p=.01 (Table 1).

**Table 1:** Demographics of the study population by the Enhanced Recovery After Surgery (ERAS) status ERAS: Enhanced Recovery After Surgery; BMI: body mass index; ASA: American Society of Anesthesiologists Continuous variables reported as mean (standard deviation (SD)) and compared with t-test; categorical variables reported as frequency (%) and compared with the chi-square test. Data are mean ±SD or n (%). ^a^Includes salpingectomy, unilateral or bilateral ovarian cystectomy, or salpingo-oopherectomy when no hysterectomy was performed. ^b^With or without salpingectomy, salpingo-oopherectomy, or other procedures including those for pelvic organ prolapse. ^c^Including any radical hysterectomies or surgeries including lymph node dissections or other staging procedures.

		Total	ERAS	Non-ERAS	p
		n=328	n=142	n=186	
Age (years)		51.0 (0.8)	50.9 (1.2)	51.1 (1.0)	0.93
BMI (kg/m^2^)		28.5 (0.4)	28.5 (0.6)	28.6 (0.5)	0.89
Surgical time (min)		251.3 (5.5)	235.5 (8.1)	263.3 (7.3)	0.01
ASA	Class 1	25 (7.6%)	9 (6.3%)	16 (8.6%)	0.56
	Class 2	194 (59.2%)	81 (57.0%)	113 (60.8%)	
	Class 3	106 (32.3%)	50 (32.2%)	56 (30.1%)	
	Class 4	3 (0.9%)	2 (1.4%)	1 (0.5%)	
History of diabetes		30 (9.3%)	14 (9.9%)	16 (8.6%)	0.70
History of chronic kidney disease		12 (3.7%)	5 (3.5%)	7 (3.8%)	0.91
History of coronary artery disease		4 (1.2%)	2 (1.4%)	2 (1.4%)	0.79
Diagnosis	Benign	241 (73.5%)	101 (71.1%)	140 (75.3%)	0.40
	Malignant	87 (26.5%)	41 (28.9%)	46 (24.7%)	
Surgical approach	Laparoscopic surgery	133 (40.6%)	61 (42.9%)	72 (38.7%)	0.44
	Robotic surgery	195 (59.4%)	81 (57.1%)	114 (61.3%)	
Surgeon	Gynecologist	151 (46.0%)	66 (46.5%)	85 (45.7%)	0.33
	Gynecologic oncologist	145 (44.2%)	66 (46.5%)	79 (42.5%)	
	Uro-gynecologist	32 (9.8%)	10 (7.0%)	22 (11.8%)	
Type of surgery	Adnexal^a^	28 (8.5%)	11 (7.8%)	17 (9.1%)	0.34
	Benign hysterectomy^b^	173 (52.7%)	74 (52.1%)	99 (53.2%)	
	Cancer hysterectomy^c^	83 (25.3%)	37 (26.0%)	46 (24.7%)	
	Myomectomy	29 (8.8%)	10 (7.0%)	19 (10.2%)	
	Others	15 (4.6%)	10 (7.0%)	5 (2.7%)	

Univariate analysis

The median total opioid use was significantly less among patients undergoing ERAS compared with patients who were non-ERAS (17.4 vs. 36.2 mg; p<0.001). While the opioid use on postoperative day 1 was significantly less in patients in the ERAS group compared to those in the non-ERAS group (p<0.001), the opioid use on postoperative day 0 did not differ by use of the ERAS protocol (p=0.24) (Table 2). Among the patients with benign diagnoses, only the median total opioid use was significantly less with ERAS compared to non-ERAS (21.1 vs. 30.8 mg; p=0.002). Among the patients with a malignant diagnosis, the patients in the ERAS group had significantly more opioid use on postoperative day 0 but less opioid use compared to the non-ERAS group on postoperative day 1. However, overall, the ERAS group experienced lower total opioid use and shorter hospital stay (p<0.01). When stratified by a surgical approach, among the patients who underwent laparoscopic surgery, the median total opioid use was significantly less with ERAS compared to non-ERAS (21.1 vs. 30.3 mg; p=0.02). Among the patients who underwent robotic surgery, the patients in the ERAS group had significantly less total opioid use and opioid use on postoperative day 1 (p<0.001). 

**Table 2:** Analgesic requirement and length of stay by the ERAS status ERAS: Enhanced Recovery After Surgery; MME: morphine milligram equivalents unit. Outcomes reported as median (interquartile range) and compared with Wilcoxon rank-sum test.

		ERAS	Non-ERAS	p
		n=142	n=186	
Total	MMEs postop day 0 (mg)	7.5 (2.4-17)	5.4 (0-17)	0.24
	MMEs postop day 1 (mg)	7.5 (0-19)	14.3 (0-30)	<0.001>
	Total MMEs (mg)	17.4 (5.8-32.5)	36.2 (15-77.2)	<0.001>
	Hospital stay (days)	1.4 (1.2-2.2)	1.5 (1.3-2.3)	0.006
Stratified by diagnosis				
Benign	MMEs postop day 0 (mg)	9.0 (2.9-17.5)	8.0 (1.6-2.45)	0.89
	MMEs postop day 1 (mg)	8 (0-22.5)	14.0 (5-25.5)	0.05
	Total MMEs (mg)	21.1 (8-34)	30.8 (15-62.9)	0.002
	Hospital stay (days)	1.2 (1.2-2.1)	1.4 (1.2-2.2)	0.28
Malignant	MMEs postop day 0 (mg)	3.5 (0-10)	0 (0-2.4)	<0.001>
	MMEs postop day 1 (mg)	5.0 (0-12)	14.3 (0-77.2)	0.01
	Total MMEs (mg)	10.8 (3.2-18.5)	72.0 (30-86.2)	<0.001>
	Hospital stay (days)	1.4 (1.3-2.2)	2.3 (2-3.3)	<0.001>
Stratified by surgical approach				
Laparoscopic surgery	MMEs postop day 0 (mg)	9.1 (3.2-17.5)	9.1 (2-23)	0.38
	MMEs postop day 1 (mg)	10.0 (0-22.5)	10.0 (0-24.5)	0.49
	Total MMEs (mg)	21.1 (9.1-33.6)	30.3 (15.1-54)	0.02
	Hospital stay (days)	1.3 (1.2-1.5)	1.4 (1.2-2.2)	0.07
Robotic surgery	MMEs postop day 0 (mg)	6.6 (1.5-15.8)	2.5 (0-10.7)	0.05
	MMEs postop day 1 (mg)	7.5 (0-15)	15.0 (3.2-35.4)	0.0002
	Total MMEs (mg)	14.0 (5.3-31.1)	44.3 (15-80.8)	<0.0001>
	Hospital stay (days)	1.4 (1.3-2.3)	2.0 (1.3-2.4)	0.05

Multivariate analysis

In multivariate analysis adjusting for age and surgical time, the ERAS protocol resulted in decreasing opioid use on postoperative day 1 by 30.9% (p=0.006) compared to patients not on the ERAS protocol, decreasing total opioid use by 58.5% (p<0.001) and shortening hospital stay by 11.3% (p=0.006) (Table 3). Stratified by diagnosis, ERAS was significantly associated with decreased total opioid use among benign diagnoses, and among malignant diagnoses, it was associated with decreased opioid use on postoperative day 1, decreased total opioid use, and shorter hospital. When comparing malignant to benign cases, opioid use on postoperative day 0 was not different by the ERAS status. However, the malignant cases showed a larger magnitude of difference between ERAS and non-ERAS compared to benign cases with regard to opioid use on postoperative day 1 and overall opioid use for their hospital stay (interaction p<0.01) (Table 3). 

**Table 3:** Associations between the ERAS status with opioid requirement and length of hospital stay among all patients and stratified by diagnosis and by surgical approach ERAS: Enhanced Recovery After Surgery; MME: morphine milligram equivalents unit; betas (SE) and p values of ERAS compared to non-ERAS are from linear regression models between the ERAS status and each outcome adjusted for age and surgical time. *interaction p value tested the difference in outcome between diagnosis of benign vs. malignant; **interaction p value tested the difference in outcome between laparoscopic vs robotic surgery.

	Total N=328		Benign N=241		Malignant N=87		Interaction p*	Laparoscopic surgery N=133		Robot-assisted surgery N=195		Interaction p**
	% change (SE)	p	% change (SE)	p	% change (SE)	p		% change (SE)	p	% change (SE)	p	
Log MMEs PO day 0 (mg)	-13.06 (12.75)	0.25	-15.63 (15.03)	.21	23.37 (34.99)	0.51	0.28	-0.29 (0.17)	0.08	0.05 (0.18)	0.80	0.16
Log MMEs PO day 1 (mg)	-30.93 (13.88)	0.006	-18.94 (16.18)	.16	-60.54 (32.31)	0.002	0.01	-0.28 (0.21)	0.19	-0.44 (0.17)	0.01	0.63
Log total MMEs (mg)	-58.52 (12.75)	<0.001>	-44.57 (15.03)	< .001>	-82.27 (24.61)	<0.001>	<0.001>	-0.47 (0.18)	0.01	-1.18 (0.16)	<0.001>	0.007
Log hospital stay (days)	-11.31 (4.08)	0.006	-4.88 (5.13)	.16	-30.93 (8.33)	<0.001>	<0.001>	-0.29 (0.17)	0.08	0.05 (0.18)	0.80	0.16

Among laparoscopic surgeries, ERAS was significantly associated with decreased total opioid use, and among robotic surgeries, ERAS was associated with decreased opioid use on postoperative day 1 and total opioid use. Patients who underwent robotic surgery showed a larger magnitude of difference between ERAS and non-ERAS compared with patients who underwent laparoscopic surgery (interaction p=0.007) (Table 3). 

Effect of the QL Block

Twenty-nine patients in the ERAS group (20.4%) did not receive a QL block but were still included as they otherwise received the ERAS protocol. Reasons for not receiving the block included patient, surgeon, or anesthesiologist declination or unavailability of an anesthesiologist to perform the procedure. Among 142 patients in the ERAS group, opioid use and length of hospital stay were similar between patients who did and did not receive the QL block (p>0.20) (Table 4). For those who received the block, there were no block-related complications, such as vascular puncture, bowel puncture, or local anesthetic toxicity.

**Table 4:** Opioid requirement and length of hospital stay by QL block status among patients receiving the ERAS protocol ERAS: Enhanced Recovery After Surgery; MME: morphine milligram equivalents unit; QL: quadratus lumborum block. Outcome reported as median (interquartile range) and compared with Wilcoxon rank-sum test.

	QL block	no QL block	p
	n=113	n=29	
MMEs postop day 0 (mg)	7.5 (1.6-17)	6.6 (2.4-16.6)	0.85
MMEs postop day 1 (mg)	8.0 (0-20)	5.0 (0-17)	0.49
Total MMEs (mg)	17.4 (7.5-33.2)	10.8 (5.6-30.8)	0.54
Hospital stay (days)	1.4 (1.3-2.2)	1.3 (1.2-1.6)	0.20

## Discussion

The present study was conducted to exhibit the effect the ERAS pathway on the perioperative course of minimally invasive gynecologic surgery patients. This is the first study to our knowledge to evaluate an ERAS protocol for patients undergoing gynecologic surgery that standardly included a QL block. The ERAS group had significant decreases in opioid use on postoperative day 1, in total admission opioid use, and hospital length of stay compared to the non-ERAS group. In the era of the opioid epidemic and increased prioritization on decreasing hospital cost, our study adds to the growing body of literature demonstrating that ERAS protocols are effective at reducing opioid requirements and length of stay among patients undergoing gynecologic surgery [15-20].

Since the introduction of this pathway internationally, it has been shown that specific components of the ERAS pathway have been associated with improved postoperative outcomes. Patient education and multimodal analgesia were vital in the implementation of our protocol and likely contributed significantly to the lower MME requirements [19]. In addition, euvolemia and early feeding are thought to contribute to a faster return of bowel function and faster discharge from the hospital [21,22].

When stratifying our data by surgical approach, ERAS patients undergoing robotic surgery had a significant decrease in postoperative day 1 MME requirements. Meanwhile, we did not observe a difference among patients undergoing laparoscopic surgery. Reasons for this are unclear but one may have to do with less incisions in laparoscopic surgery (sometimes 1 vs. 5). There also may be selection bias with more complex and extensive surgeries being performed robotically. These differences in incisions and the extent of dissection may result in robotic cases showing more benefit from a multimodal approach.

While our data show that the ERAS protocol benefitted all our patients, it particularly benefitted patients with malignant diagnoses compared to benign diagnoses. Patients with malignant diagnoses had decreases in total opioid use and opioid use on postoperative day 1 along with a shorter hospital stay. The median hospital stay among cancer patients in the non-ERAS group was almost a full day longer than in the ERAS group, and their total MME requirements were dramatically higher. An explanation for this may be that cancer surgeries often require additional procedures, such as lymphadenectomies or radical dissection. The implementation of a formalized protocol may have thus more greatly impacted the cancer patients than those with a benign diagnosis who already were expected to have a somewhat faster recovery.

The QL block was implemented as a part of our multimodal analgesia aspect of the ERAS pathway to potentially further reduce our patients’ opioid requirements. Two randomized controlled trials among women undergoing laparoscopic hysterectomy showed that a QL block decreased postoperative opioid requirements and time to first opioid use compared to women receiving either placebo or a TAP block [10,13,14,23]. While the dual effect of the QL blocks on somatic and visceral pain in theory should improve analgesia, our patient population did not appear to benefit from them. As the ERAS pathway becomes a mainstay with our gynecologic surgeries, one of our future endeavors will be to further delineate the effect of the QL.

The limitations of this study include that it was retrospective in nature. In addition, there was a significantly shorter operative time among the patients in the ERAS group compared to non-ERAS. The faster ERAS group surgeries may reflect changes in learning curves or practice patterns over time. For example, a shift in gynecologic oncology surgeries from performing full lymphadenectomies to more sentinel lymph node dissections also occurred during this time and results in shorter surgeries [24,25]. In addition, we were unable to collect data on compliance to the protocol or on other specific outcomes, such as nausea, time to ambulation, tolerating oral intake, or patient satisfaction. However, the data are convincing enough to show that if a protocol is in place, perioperative outcomes are significantly improved even if not all components were met. Finally, excluding patients with a laparotomy and chronic opioid use also limits the generalizability of this protocol to this group of patients.

## Conclusions

ERAS implementation in the minimally invasive gynecologic surgery population is associated with a significant reduction in postoperative opioid requirements and length of hospital stay, and this reduction was most dramatic among patients undergoing robotic gynecologic cancer surgeries. By continuing to improve our perioperative care, we can further reduce opioid use and prescriptions while improving patient satisfaction and perioperative outcomes. Overall, our findings support the adoption of ERAS protocols in minimally invasive gynecologic surgery.

## Figures and Tables

**Figure 2:**
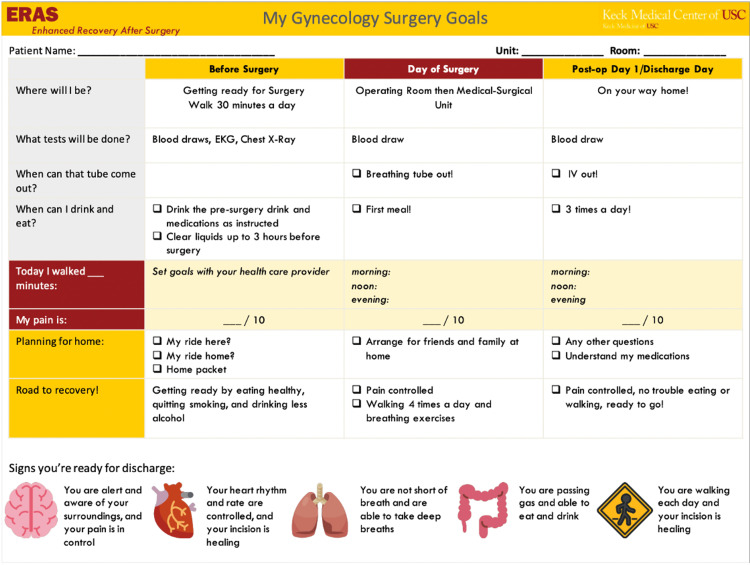
Example of Keck Medicine of USC ERAS Surgery Goals

**Figure 3:**
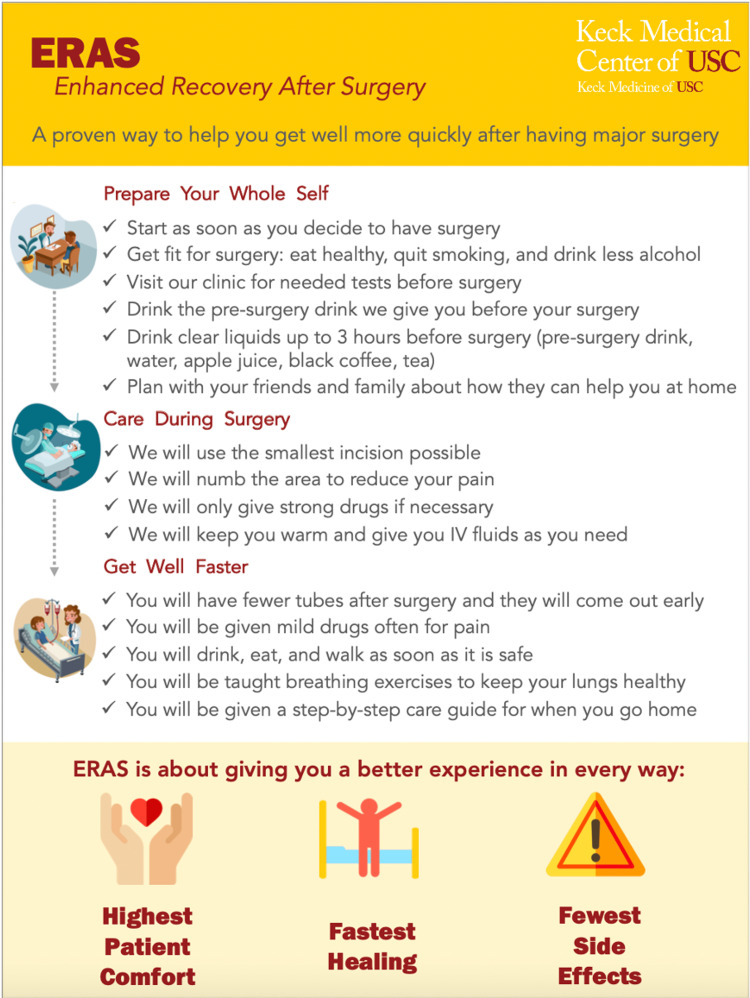
Example of Keck Medicine of USC ERAS Patient Infographic

## Appendices

Appendix 1. Example Gynecology ERAS Protocol

*Preoperative* 

1.     Surgery scheduling should list patient as an “(ERAS) patient” to identify ERAS patients.

2.     Pre-op timeline:

a.     As soon as possible after surgery is planned but at least ONE to TWO weeks prior to elective surgery: all patients should be seen in Pre-Op clinic

                                               i.     Obtain CBC, CMP (including prealbumin), Type and Screen 

                                              ii.     Encourage preoperative health optimization 

1.     Walking 30 minutes a day. Patients who are unable to walk for 30 minutes should be encouraged to gently increase exercise level within their limits 

2.     Working out 30 minutes a day

3.     Nutrition optimization

4.     Smoking and alcohol cessation 

                                             iii.     All patients to receive ERAS Handbook 

b.     No routine bowel prep 

c.     Encourage to take carbohydrate drink and clear liquids up until 2 hours before surgery. 

Day of Surgery

1.     Chronic Pain Patients should take their home pain medications the morning of surgery as discussed at Pre-Op clinic. 

2.     Type and Cross - per surgery

3.     Pre-op medications: ordered by anesthesia resident and CRNA - e.g. in pre-surgery area with small sips of water

a.     Gabapentin 300 mg 

b.     Celecoxib 200 mg (decrease dose to half in patients with cirrhosis [Child-Pugh Class B] & avoid in pts with sulfa allergy, h/o CAD) 

c.     Acetaminophen 1000 mg 

*Intraoperative* 

1.     Medications: 

a.     Follow perioperative antibiotic dosing recommendations 

b.     Quadratus Lumborum Block - 20 cc 0.25% Bupivacaine + 10 cc of liposomal bupivacaine per side prior to induction

c.     Dexamethasone 4 mg IV (discuss first with surgical team if the patient is diabetic)

d.     Ketamine 0.2 mg/kg bolus

e.     TIVA 

                                               i.     Propofol infusion 100-150mcg/kg/min 

                                              ii.     Ketamine 10mg/hr (via boluses or infusion)

                                             iii.     Dexmedetomidine 0.2-0.7 mcg/kg/hr

f.      Ketorolac 30 mg at end of case (be aware of kidney function and bleeding risk) 

                                               i.     If patient weighs < 50 kg, give 15 mg (do not give Ketorolac if surgery is less than 6 hours and patient received Celecoxib preoperatively)

g.     Ondansetron 4mg

2.     Fluids: prefer Lactated Ringer’s or Plasmalyte - goal-directed restrictive, avoid liberal fluids. 

a.     Consider use of noninvasive Cardiac Monitoring - ClearSight 

3.     No routine arterial line 

4.     Laboratory goals: 

a.     Glucose goal between 110-180 throughout the surgery 

                                               i.     Check baseline glucose post induction with POC Glucose 

                                              ii.     POC Glucose Q1 Hour

b.     If sugar is not controlled within 2 hours of first check, consider starting an insulin drip 

c.     If arterial line is placed, ABG to be checked at baseline on all patients

d.     Foley catheter - order to discontinue per Surgery Team 

Appendix 2 
